# Hypothalamic c-Fos expression changes during late chicken embryonic development and increases following experimental stimulation

**DOI:** 10.1038/s41598-026-65626-x

**Published:** 2026-09-05

**Authors:** Leonhard Kowalczyk, Tobias Kowalczyk, Yile Huang, Maik Hintze, Longfei Cheng, Bernd Hilgers, Stefanie Kuerten, Qin Pu, Inga Tiemann, Ruijin Huang

**Affiliations:** 1 Institute of Neuroanatomy, University of Bonn and University Hospital Bonn, Bonn, Germany; 2 Precision Livestock Farming, Faculty of Agricultural Sciences and Landscape Architecture, Osnabrueck University of Applied Sciences, Osnabrück, Germany; 3 Institute of Animal Science, University of Bonn, Bonn, Germany

**Keywords:** Stimulation evoked responses, Hypothalamus, Chicken embryos, c-Fos, Developmental biology, Neuroscience, Physiology

## Abstract

The timing of functional hypothalamic responsiveness during embryogenesis remains incompletely defined. We examined hypothalamic activation in chicken embryos from embryonic day (ED) 12–17 following experimental stimulation consisting of a localized foot injection of paraformaldehyde (PFA) together with the associated handling procedures required for its administration. Hypothalamic activation was quantified as the proportion of c-Fos-positive cells within the paraventricular nucleus (PVN). Generalized linear modelling revealed significant effects of both embryonic age and stimulation condition on c-Fos expression, with no evidence of an Age × Stimulation interaction. Across development, stimulated embryos exhibited higher proportions of c-Fos-positive cells than age-matched controls. Independent of stimulation, the only significant difference between adjacent developmental stages occurred between ED14 and ED15, suggesting a candidate transition in hypothalamic maturation. Exploratory analysis indicated that these findings are consistent with an early (ED12–ED14) and a late (ED15–ED17) developmental phase and suggest that ED14–ED15 may represent a candidate phase boundary in hypothalamic maturation.

**Supplementary Information:**

The online version contains supplementary material available at https://doi.org/10.1038/s41598-026-65626-x.

## Introduction

The functional maturation of neural circuits during embryonic development is a gradual and highly coordinated process^1,2^. Although structural development of major brain regions has been extensively characterized across vertebrate species, the timing at which these structures acquire functional responsiveness to sensory input remains incompletely defined^3,4^. In particular, the developmental onset of central responsiveness to noxious stimulation has attracted increasing attention, as it provides insight into the maturation of sensory integration and stress-regulatory systems^5,6^.

Nociceptive processing develops hierarchically, involving peripheral sensory neurons, ascending brainstem pathways, subcortical integrative centers, and ultimately higher-order cerebral circuits^7–10^. Experimental studies across species indicate that early responses to noxious input are mediated primarily by subcortical and brainstem structures, with cortical integration emerging later in development^5^. Functional activation markers such as c-Fos are widely used to map stimulus-induced neuronal recruitment and to delineate the maturation of central circuits^11,12^. In rodents, stimulus-induced Fos expression in central nociceptive pathways increases postnatally, reflecting progressive circuit maturation^6^. These findings suggest that the acquisition of central responsiveness is not instantaneous but instead follows distinct developmental stages.

Among subcortical structures, the hypothalamus occupies a pivotal position in coordinating autonomic and neuroendocrine responses to stress-related and sensory stimuli^13^. The paraventricular nucleus (PVN) integrates ascending sensory input with endocrine and autonomic outputs and is a key regulator of hypothalamic–pituitary–adrenal (HPA) axis activation^14,15^. While the structural development of hypothalamic nuclei has been described in multiple species^16^, considerably less is known about the developmental timing of functional hypothalamic activation in response to peripheral noxious stimulation. Defining this timing is essential for understanding the ontogeny of central stress and sensory processing systems.

Recent studies in avian embryos have begun to characterize neural and physiological reactivity during late *in ovo* development. Electrophysiological recordings indicate that organized EEG activity becomes detectable during the second half of incubation, suggesting increasing neuronal coordination within the developing brain^17–19^. In parallel, behavioural and cardiovascular responses to noxious mechanical stimulation have been reported during late embryogenesis^20,21^. However, these approaches provide either global measures of neuronal activity or system-level physiological outputs and do not allow precise identification of region-specific neuronal recruitment. Consequently, the development of hypothalamic activation in response to noxious stimulation has not yet been characterized with cellular resolution in the avian embryo.

In the present study, we investigated the effects of embryonic age and experimental stimulation on hypothalamic c-Fos expression in chicken embryos between embryonic day (ED) 12 and ED17. C-Fos expression within the PVN was quantified following an experimental stimulation comprising a localized paraformaldehyde (PFA) injection and the associated handling procedures. By examining age-related variation and the response associated with experimental stimulation, we provide insight into the temporal dynamics of subcortical functional maturation during late embryonic development.

## Results

To investigate the developmental timing of hypothalamic activation in response to external stimulation, chicken embryos from ED12 to ED17 were examined. Embryos assigned to the experimental group underwent the stimulation procedure and were perfused 90 min later, corresponding to the reported peak of forebrain c-Fos expression. Control embryos were collected and perfused immediately after removal from the egg without undergoing the stimulation procedure. Consequently, compared with controls, embryos in the experimental group were exposed to both the local injection procedure and the associated handling required for its administration.

**Fig. 1:**
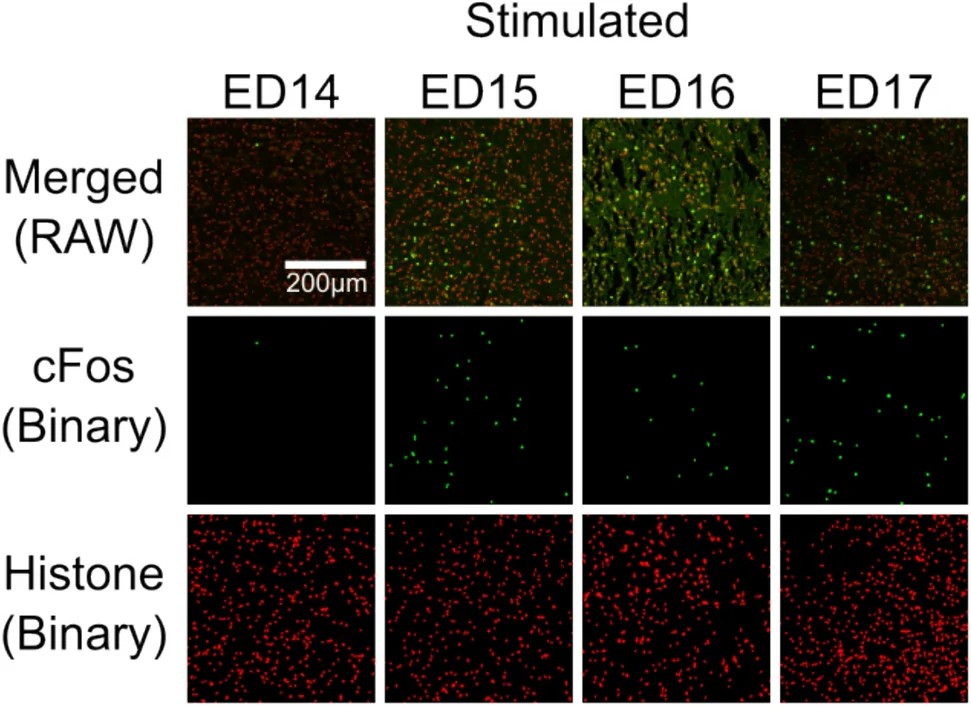
Representative immunofluorescence images of the paraventricular nucleus (PVN) in stimulated embryos across embryonic days ED14–ED17. "Merged (raw)" shows the unprocessed fluorescence images, whereas binary images of c-Fos (green) and Histone H3 (red) were generated by FIJI during image post-processing. Histone H3 staining exhibited a consistent distribution across developmental stages and treatment groups. Representative images for ED12–ED13 are shown in Fig. S2. Section size: 464.98 × 464.98 µm; scale bar: 200 µm.

C-Fos-positive cells were identified by immunohistochemistry in coronal sections containing the paraventricular nucleus (PVN) of the hypothalamus (Fig. 1; Figs. S1–S2). Hypothalamic activation was quantified as the proportion of c-Fos-positive cells within the PVN. Cell counts were obtained using Histone H3 immunostaining to identify all nuclei. Cell detection and quantification were performed using an automated FIJI-based image analysis pipeline^22^. Representative examples of c-Fos and Histone H3 immunostaining across developmental stages are shown in Fig. 1. The resulting proportion of c-Fos-positive cells was used as a quantitative measure of hypothalamic activation for all subsequent analyses.

### Experimental stimulation is associated with higher c-Fos expression across embryonic development

To evaluate the effects of embryonic age and experimental stimulation on hypothalamic c-Fos expression, the proportion of c-Fos-positive cells was analysed using a quasibinomial generalized linear model (GLM) with age group (ED12–ED17), stimulus condition (control vs. stimulated), and the Age × Stimulus interaction included as fixed effects. Analysis of deviance revealed significant main effects of both age group (F (5, 65) = 13.06, p < 0.001) and stimulus condition (F (1, 65) = 123.4, p < 0.001; Supplementary Table S01), indicating that c-Fos expression was increased across developmental stages and following experimental stimulation.

**Fig. 2:**
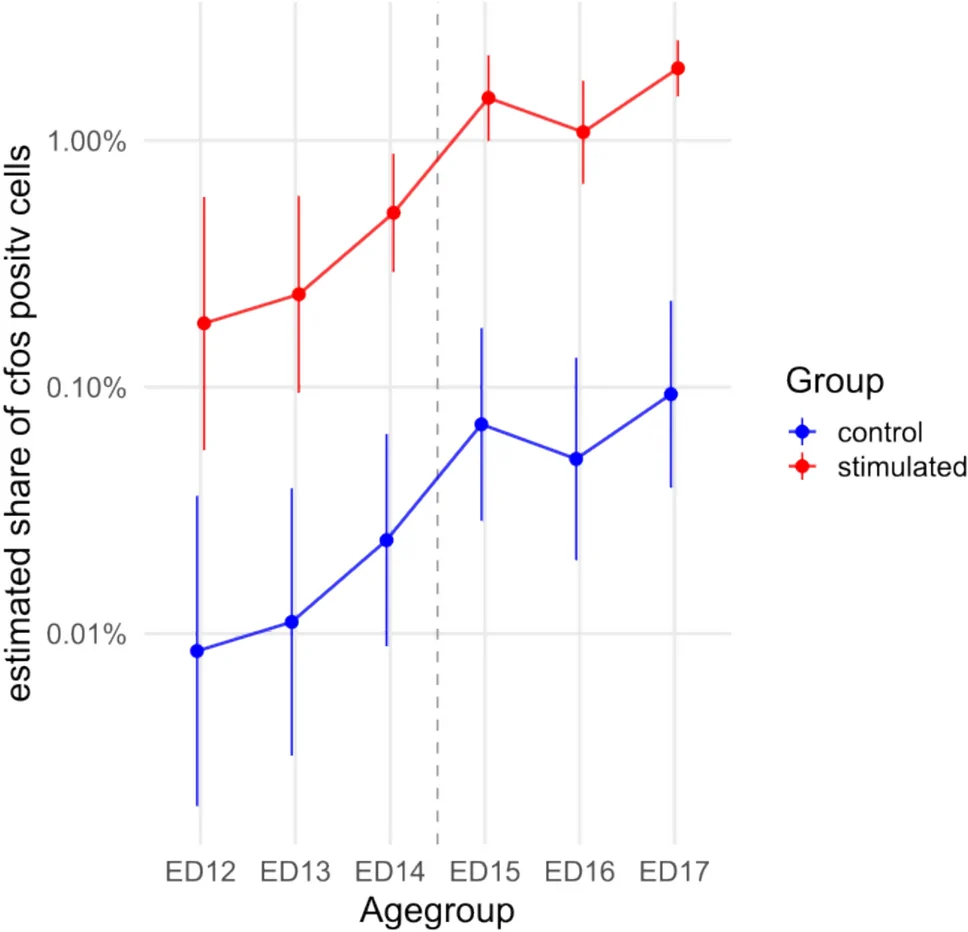
c-Fos expression varies across embryonic development and with experimental stimulation condition. Estimated marginal means derived from the quasibinomial generalized linear model (GLM) are shown for the proportion of c-Fos-positive cells across developmental stages (ED12–ED17) and according to stimulation condition. Dashed vertical line indicates the ED14/ED15 boundary. Error bars indicate 95% Cis. Source data are provided as a Source Data file.

The effect of stimulation was consistently positive across the developmental period examined, with stimulated embryos exhibiting higher estimated proportions of c-Fos-positive cells than age-matched controls (Fig. 2; Fig. S3; Supplementary Table S02). No significant Age × Stimulus interaction was detected (F (5, 60) = 0.15, p = 0.98; Supplementary Tables S03, S04), indicating that the magnitude of the stimulation effect did not differ significantly among age groups. Thus, while overall c-Fos expression changed during development, the relative increase associated with stimulation remained comparable across embryonic ages.

### Developmental increase in c-Fos expression identifies a transition between ED14 and ED15

**Fig. 3:**
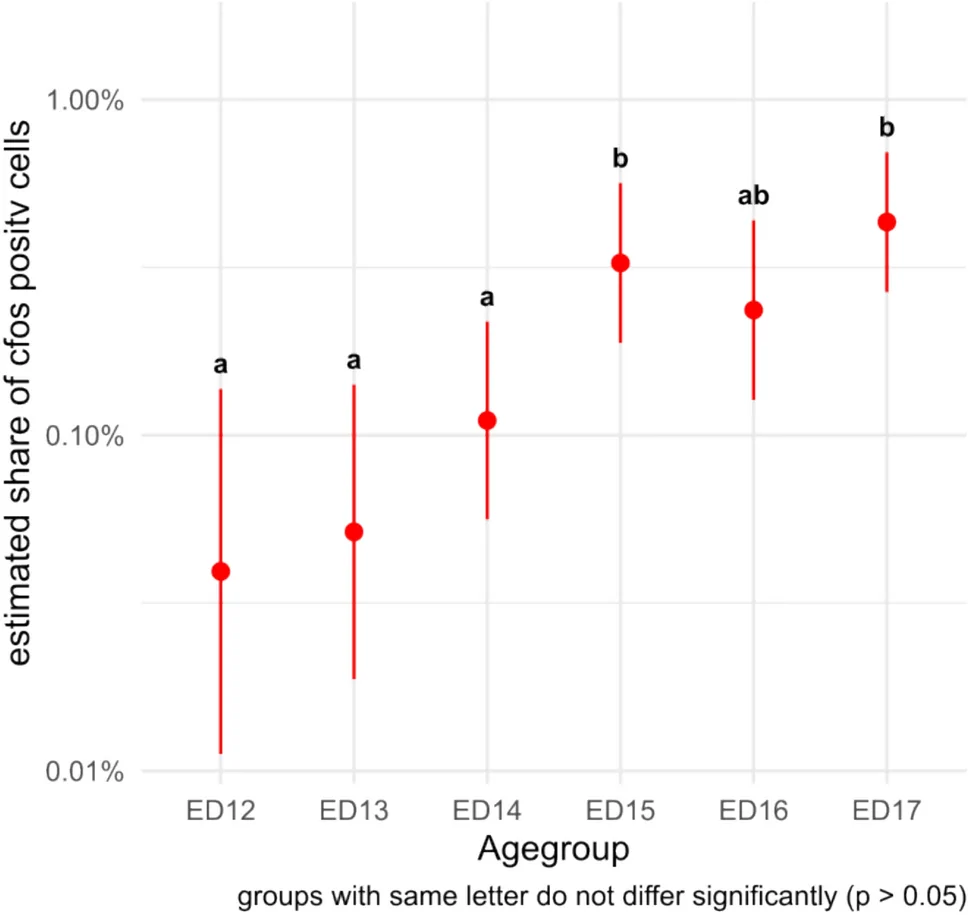
Two distinct developmental phases of c-Fos expression across embryonic age. Pairwise comparisons were performed using Tukey-adjusted estimated marginal means derived from the quasibinomial GLM, marginalised over stimulus condition. Compact letter display summarises pairwise comparisons. ED12–ED14 are grouped into a lower-expression cluster (**a**) and ED15 – ED17 into a higher-expression cluster (**b**). No significant differences were detected among ages within each cluster. ED16 (ab) did not differ significantly from either cluster. Error bars indicate 95% CIs. Source data are provided as a Source Data file.

To further characterize the significant effect of embryonic age on c-Fos expression, Tukey-adjusted post hoc pairwise comparisons were performed between all age groups based on estimated marginal means derived from the quasibinomial GLM (Fig. 3; Supplementary Tables S05, S06). These comparisons revealed a distinct developmental pattern in which embryos from ED12–ED14 generally exhibited lower proportions of c-Fos-positive cells, whereas embryos from ED15–ED17 exhibited higher proportions. Consistent with this pattern, compact letter display analysis grouped ED12–ED14 into a lower-expression cluster and ED15–ED17 into a higher-expression cluster. ED16 occupied an intermediate position and did not differ significantly from either cluster (Fig. 3).

To identify the timing of the developmental transition more specifically, pairwise comparisons between adjacent age groups were examined. Among all neighbouring developmental stages, a significant increase in c-Fos expression was detected only between ED14 and ED15 (p = 0.028), whereas no significant differences were observed between ED12 and ED13, ED13 and ED14, ED15 and ED16, or ED16 and ED17. This finding suggests that the principal age-related change in c-Fos expression occurred between ED14 and ED15 and identifies this interval as a candidate developmental transition (Supplementary Table S05).

### Exploratory two-phase analysis of c-Fos expression

The age-group comparisons suggested that c-Fos expression was organized into two developmental phases, with lower expression levels observed from ED12–ED14 and higher expression levels from ED15–ED17. To explore whether this age-related pattern could be represented by two developmental phases, a series of two-phase models was fitted (Supplementary Table S07). In each model, embryos were grouped into an “early” and a “late” developmental phase using one of the five possible age-group boundaries (ED12/13, ED13/14, ED14/15, ED15/16, or ED16/17), and model fit was evaluated using residual deviance.

Among all candidate models, the transition point between ED14 and ED15 yielded the lowest residual deviance (1008.5; Supplementary Table S07), indicating the best fit to the observed data. This result was consistent with the post hoc age-group comparisons, which identified the ED14–ED15 interval as the only significant difference between adjacent developmental stages.

**Fig. 4:**
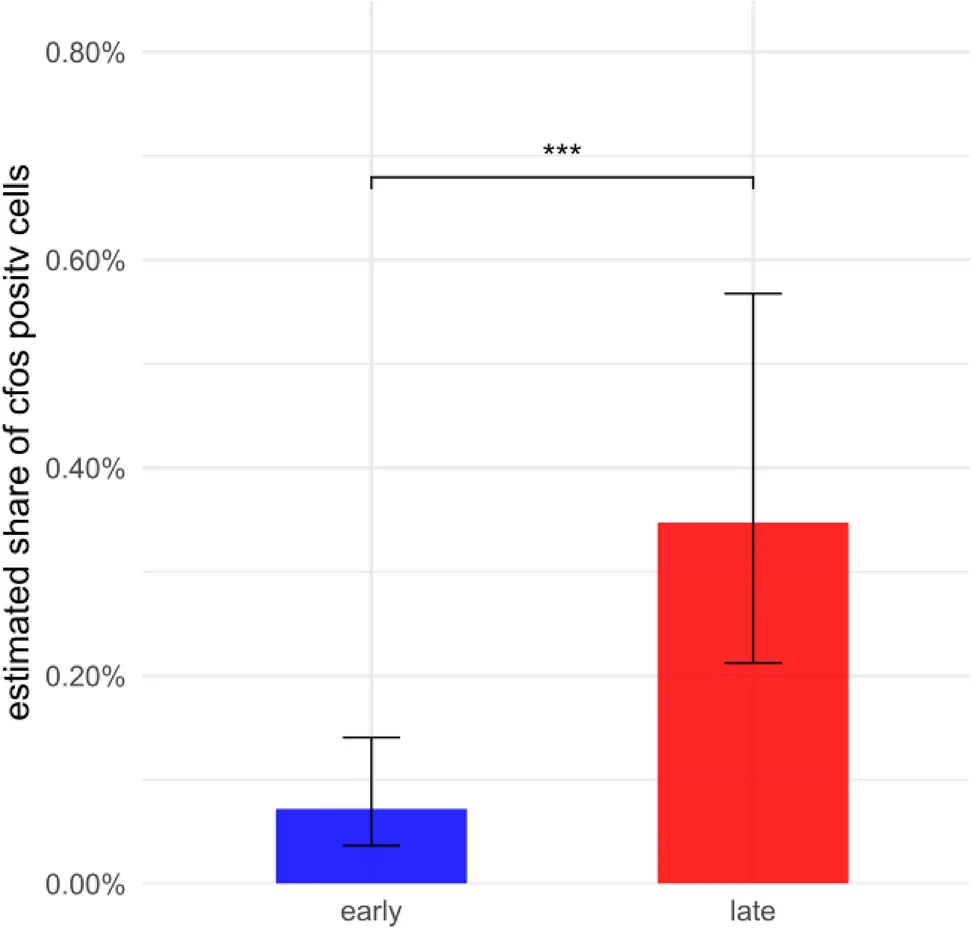
Exploratory two-phase representation of c-Fos-positive cells. Embryos were grouped into an early (ED12–ED14) and a late (ED15–ED17) developmental phase. The proportion of c-Fos-positive cells differed significantly between the two groups (F(1,69) = 44.04, p < 0.001). Statistical comparisons were based on estimated marginal means derived from the two-phase quasibinomial GLM (Phase + Stimulus). Error bars indicate 95% CIs. Source data are provided as a Source Data file.

To determine whether the simplified two-phase representation adequately captured the age-related variation in c-Fos expression, the best-fitting phase model was compared with the full model in which each embryonic day was treated as a separate age group. The two models did not differ significantly (F (4, 65) = 2.36, p = 0.063; Supplementary Table S08), indicating that the additional complexity of the full six-age-group model did not substantially improve model fit. Thus, the developmental pattern is consistent with a two-phase representation, with ED14–ED15 representing the best-supported candidate transition among the models tested (Fig. 4).

### No evidence that the exploratory phases pattern differs by stimulus condition

To determine whether the developmental transition identified between ED14 and ED15 depended on stimulus condition, the best-fitting two-phase model (ED12–ED14 vs. ED15–ED17) was extended to include stimulus condition and a Phase × Stimulus interaction term. This analysis tested whether the magnitude of the developmental shift differed between control and stimulated embryos.

In this exploratory model, both developmental phase and stimulus condition were associated with c-Fos expression (Phase: F (1, 68) = 43.51, p < 0.001; Stimulus: F (1, 68) = 100.44, p < 0.001; Supplementary Table S09), indicating that embryos in the later developmental phase exhibited higher c-Fos expression overall and that experimental stimulation was associated with increased c-Fos expression across developmental stages.

In contrast, there was no evidence of a Phase × Stimulus interaction (F (1, 68) = 0.04, p = 0.847; Supplementary Table S09), indicating that the magnitude of the developmental phase shift did not detectably differ between control and stimulated embryos. Thus, the developmental increase in c-Fos expression and the effect of stimulation showed separate associations with the observed variation in c-Fos-positive cell proportions.

## Discussion

The present study demonstrates that both embryonic age and experimental stimulation influence hypothalamic c-Fos expression during late chicken embryogenesis. Across all developmental stages examined, stimulated embryos exhibited higher proportions of c-Fos-positive cells than age-matched controls, indicating that the experimental stimulation was associated with increased hypothalamic activation. Independent of stimulation, c-Fos expression showed its only significant difference between adjacent developmental stages between ED14 and ED15. This pattern was consistent with a developmental transition occurring during this interval. Moreover, it was well described by a two-phase model comprising an early developmental phase (ED12–ED14) and a later phase (ED15–ED17). Notably, there was no evidence that the age-related pattern differed between groups, suggesting that it may reflect an intrinsic developmental change in hypothalamic activity rather than a stimulus-specific effect.

External sensory stimulation rapidly induces expression of the immediate early gene c-Fos, whose protein product regulates downstream genes involved in neuronal plasticity and adaptation^23,24^. Accordingly, c-Fos is widely used as a marker of recent neuronal activation^25^. The increasing c-Fos responsiveness observed in the embryonic PVN therefore likely reflects progressive maturation of hypothalamic networks that enables embryos to integrate external sensory information and generate adaptive neuroendocrine responses^26,27^.

Independent of stimulation condition, c-Fos expression within the PVN showed its only significant difference between adjacent developmental stages between ED14 and ED15. Together with the two-phase model analysis, this suggests that ED14–ED15 represents a candidate developmental transition. Because this transition occurred in both control and stimulated embryos, it likely reflects an intrinsic maturational change in hypothalamic activity rather than a stimulus-specific effect.

The resulting two-phase pattern (ED12–ED14 versus ED15–ED17) suggests a candidate developmental shift around ED14/ED15 in activity across late embryogenesis. Such transitions are characteristic of neural maturation and may reflect ongoing synaptogenesis, circuit refinement, and the establishment of functional connectivity within hypothalamic networks^1,2,28–30^.

Several neurodevelopmental mechanisms may underlie this transition. Late embryogenesis is characterized by increased synaptogenesis and structural circuit formation^1,31^, alongside activity-dependent refinement of afferent projections^32^. The PVN serves as a central integrative hub for autonomic and neuroendocrine stress responses^13,33^, and its maturation enables coordinated central activation. The marked increase in c-Fos expression between ED14 and ED15 likely reflects effective recruitment of ascending sensory pathways together with consolidation of intrinsic hypothalamic connectivity^7,8^. Thus, the relatively higher c-Fos expression observed from ED15 onward may represent the attainment of sufficient synaptic integration within hypothalamic networks, consistent with developmental models in which structural maturation precedes discrete functional transitions^34^.

The relatively low c-Fos expression observed at ED16 is unlikely to represent a technical artefact, as it was present in both control and stimulated embryos. Instead, this pattern may reflect a transient developmental state of hypothalamic network activity. Around this stage, ongoing maturation and reorganization of neural circuits may temporarily alter neuronal responsiveness or the coupling between external stimulation and c-Fos induction. This interpretation is further supported by similar age-related changes observed in our EEG recordings, suggesting that ED16 may represent a distinct functional transition within late embryonic development. However, because this observation was not the primary focus of the present study, it should be interpreted cautiously and investigated further in future experiments.

Across all developmental stages examined, stimulated embryos exhibited higher proportions of c-Fos-positive cells than age-matched controls. Importantly, the applied stimulation combined a localized PFA injection with the unavoidable handling procedures required for its administration. The observed increase in stimulated embryos therefore likely reflects a response to a complex aversive stimulus rather than to nociceptive input alone. Nevertheless, the consistent difference between stimulated and control embryos across developmental stages indicates that the experimental stimulation elicited additional hypothalamic activation beyond baseline activity.

Notably, the absence of a significant Age × Stimulus interaction indicates that the age-related pattern in c-Fos expression was independent of stimulation condition. Thus, while stimulation was associated with elevated c-Fos expression across development, there was no evidence that it altered the underlying age-related pattern. These findings support the interpretation that late embryonic maturation of hypothalamic activity and responsiveness to external stimulation represent related but distinct processes.

The candidate developmental transition around ED14 and ED15 identified in the present study coincides with other reported indicators of neural maturation in the chicken embryo. Meaningful EEG activity emerges around ED13^19^, suggesting increasing functional organization of embryonic neural circuits. The timing of this transition also broadly corresponds with the appearance of behavioural and physiological responses to external stimulation, including consistent nocifensive movements from approximately ED15^20^ and significant cardiovascular responses from ED16 onward^21^. Although these measures reflect different levels of neural organization, their temporal proximity suggests coordinated maturation of central integrative circuits and their downstream effector systems during late embryogenesis.

Several limitations should be considered when interpreting these findings. First, the experimental stimulation consisted of both a localized PFA injection and associated handling procedures, preventing definitive attribution of the observed responses to nociceptive input alone. Second, c-Fos expression provides an indirect marker of neuronal activation and does not distinguish between specific sensory modalities or functional pathways. Finally, the present study focused exclusively on hypothalamic activation and does not address the developmental status of telencephalic or higher-order cerebral circuits. Consequently, the findings suggest a developmental transition in subcortical hypothalamic activity but do not establish when more complex cerebral processing becomes functionally operative. Future studies combining region-specific neural mapping with electrophysiological approaches will be required to characterize how maturation of hypothalamic activity relates to the development of broader forebrain networks.

## Conclusion

In conclusion, the present study demonstrates that hypothalamic c-Fos expression varies during late chicken embryogenesis and exhibits its only significant adjacent-age difference between ED14 and ED15. Exploratory two-phase analysis identified ED14/ED15 as a candidate phase boundary, with no evidence that the age-related pattern differed between stimulation conditions. Experimental stimulation was associated with higher levels of c-Fos expression than those observed in age-matched controls across development. Together, these findings provide exploratory evidence that the ED14–ED15 interval represents a candidate period of hypothalamic maturation during late embryogenesis. However, because the stimulation paradigm combined a localized PFA injection with associated handling procedures, the observed responses cannot be attributed exclusively to nociceptive input. Future studies employing refined stimulation paradigms will be necessary to clarify the contribution of nociceptive signaling to hypothalamic activation during embryonic development.

## Methods

### Animals

Fertilised Ixworth eggs (Gallus gallus domesticus) were obtained from the Campus Frankenforst of the Faculty of Agricultural, Nutritional and Engineering Sciences (University of Bonn). The eggs were incubated in a forced-air Incubator (HEKA-Brutgeräte, Rietberg, Germany) at 37,8° C, 55% humidity. Incubation time was calculated from the start of incubation.

Experiments were conducted between embryonic day 12 and 17 (ED12 – ED17). On the day of experimentation, all procedures were conducted within a time window of 7 hours relative to the initial incubation time, to minimize developmental variability.

### Stimulation

For the stimulated group, 5 µl of 4 % paraformaldehyde (PFA) was injected in the sole of the left foot of the fetus. Subcutaneous injection of formalin is a well-established method for inducing chemically mediated noxious stimulation^6,35–39^. While various concentrations have been reported^6,35–40^, similar results have been demonstrated by using PFA^41^. We choose 4 % PFA, due to its efficacy in preliminary trials and availability in our laboratory.

Eggs were fenestrated at the air cell, which was localized using transmitted light and marked with a pencil. The eggshell was removed using Splinter Forceps by Feilchenfeld. All preparation procedures, including membrane opening and injection, were performed under dental loupes with an integrated light source (StarMed iMag XS Titan with Starlight Nano X, starMed GmbH & Co. KG). Eggs remained at room temperature during the procedure, which was completed within 10 min before reincubation.

The shell membrane was moistened with Locke’s solution and carefully removed using Dumont #5 forceps (No. 11251-20, Fine Science Tools GmbH). The chorioallantoic membrane was opened with Vannas–Tübingen spring scissors (No. 15003-08, Fine Science Tools GmbH) and Dumont #5 forceps while avoiding major blood vessels. The amnion was then opened, and the embryo was gently rotated using a flame-polished glass pipette to expose the left foot. The left foot was carefully exteriorized and stabilized with Dumont #5 forceps to facilitate accurate injection.

A volume of 5 µL of 4% PFA was manually injected into the foot sole over approximately 5 s using an insulin syringe (BD Micro-Fine+ U100 Demi, 0.3 × 8 mm; Becton Dickinson GmbH). Five seconds after injection, the injection site was visually inspected for leakage. Any small amount of PFA remaining on the skin surface was immediately removed with cellulose swabs (Askina® Brauncel®, B. Braun SE) before the embryo was returned to the egg. The egg was sealed with parafilm and reincubated in an upright position for 90 min to allow peak forebrain c-Fos expression to develop.

The normal control was left untreated to establish a physiological baseline. By using untreated controls, we ensured a clean physiological baseline to isolate the effect of the aversive stimulus, including chemical and mechanical components.

### Immunohistochemistry (IHC)

Transcardial perfusion was performed with 40 mL phosphate-buffered-saline (PBS) followed by 20 ml 4% PFA. Brains were post-fixed in 20 ml of 4% PFA at 4°C overnight with slight agitation. After fixation brains were rinsed with PBS and quenched in 1M NH_4_Cl in PBS (30 min, 4°C). Subsequently, brains were washed four times in PBS (30 min, 4°C) and stored in 70 % ethanol at 4°C until embedding.

Brains were automatically embedded in paraffin, oriented with telencephalon and cerebellum in the same plane, to achieve a consistent orientation, as described by Puelles et al.^42^. Coronal sections (10 µm thick) of the Hypothalamus were cut using a microtome. Sections were mounted on SuperFrost Slides, deparaffinized and rehydrated. Antigen retrieval was performed with Tris-EDTA Buffer (pH 9.0) in a 90°C water bath.

Sections were blocked with 10% fetal bovine serum (FBS) in PBS for one hour at room temperature (RT). Primary antibody incubation was performed overnight at 4°C: mouse anti-c-Fos antibody (E-8, sc-166940, Santa Cruz Biotechnology Inc., 1:500) and rabbit anti-Histone H3 (ab32356, Abcam, 1:2000) in 2%FBS in PBS. Following primary incubation, sections were washed with PBST (PBS + 0.1 % Tween 20) and incubated for one hour at RT with secondary antibody: goat anti-mouse Alexa Fluor 488 (ab150113, Abcam, 1:1000) and donkey anti-rabbit Cy3 (711-165-152, Jackson ImmunoResearch Inc., 1:1000) in 2 % FBS in PBS.

To reduce background autofluorescence, sections were stained with Sudan Black for 10 minutes. After rinsing ten times with distilled water sections were mounted with coverslip using aqueous mounting medium (Fluoroshield, ab104139, abcam).

### Histological analysis

Multiple sections per sample were imaged, using Nikon A1 Confocal Laser Scanning Microscope (LSM) at 20x magnification. Images containing the paraventricular nucleus of the hypothalamus (PVN) were selected for further analysis using FIJI^22^ (Supplementary Table S13). The PVN of one hemisphere was delineated as a Region of Interest (ROI) based on morphological structures and consistent c-Fos expression patterns that accrue independently of experimental stimulation. Because c-Fos and Histone H3 immunostaining labels only cell nuclei, magnocellular and parvocellular neurons could not be distinguished morphologically. Therefore, all nuclei within the anatomically defined PVN were included in the analysis.

To quantify c-Fos-positive cells, the Yen thresholding algorithm in FIJI was applied on the entire hemisphere, to create a binary mask of the c-Fos channel. This mask was further processed using a median filter (radius = 3) and watershed transformation, to separate overlapping nuclei. Particles within the ROI with a surface area larger than 25 pixels were counted.

For the general cell count, the Default thresholding algorithm in FIJI was used in a similar manner to create a binary mask of the Histone H3 channel. Following the same post-processing steps, particles with a surface area larger than 25 pixels within the ROI were counted. The relative proportion of c-Fos-positive cells within the ROI was calculated as:

$$Relativ cFos count [\%]=\left(\frac{Number of cFos positiv cells}{Total number of cells \left(Histone H3\right)}\right)\times 100$$

Each sample was labelled with their respective embryonic day (ED) and their treatment group (Stimulated: stim; Normal Control: ctrl) (Supplementary Table S10).

### Statistical analysis

All statistical analyses were performed in R (version 4.5.2) (Supplementary Table S13). Raw immunohistochemical cell counts were analysed at the embryo level using generalized linear models with a quasibinomial error distribution and logit link function. The response was specified as the number of c-Fos-positive together with the total number of Histone H3-positive cells, thereby modelling the proportion of c-Fos-positive cells. This approach accounted for

variation in the total number of cells counted per embryo and for overdispersion relative to the binomial distribution.

#### Model selection

The initial model included embryonic day as a categorical factor, stimulation condition, and their interaction. The interaction was assessed by comparing the full interaction model with a reduced additive model using an analysis-of-deviance F-test to account for quasibinomial overdispersion. There was no evidence that the effect of stimulation differed among embryonic days (F (5,60) = 0.15, p = 0.98; Supplementary Tables S03, S04); therefore, the interaction term was omitted, and the more parsimonious additive model containing the main effects of embryonic day and stimulation condition was used for subsequent estimation and inference. The estimated dispersion parameter was φ = 15.14.

#### Main effects

The main effects of embryonic day and stimulation condition in the final additive model were assessed using Type II analysis-of-deviance F-tests. Estimated marginal means and corresponding 95% confidence intervals were calculated, back-transformed to the response scale and reported as estimated proportions of c-Fos-positive cells. Inference was based on a t-distribution using the residual degrees of freedom (df = 65) to account for uncertainty associated with the estimated quasibinomial dispersion (Supplementary Table S01, S11).

#### Post hoc comparisons

Pairwise contrasts among the six embryonic-day groups were performed using estimated marginal means from the additive model, marginalised over stimulation condition. All 15 pairwise age-group comparisons were treated as a single family and adjusted using the Tukey method to control the family-wise error rate. Results were summarised using a compact letter display, in which groups sharing a letter showed no statistically significant difference at α = 0.05 (Supplementary Tables S05, S06).

#### Exploratory phase analysis

To explore whether the age-related pattern in c-Fos expression could be represented by two developmental phases, embryonic day was dichotomised into early and late phases at each of the five possible boundaries: ED12/ED13, ED13/ED14, ED14/ED15, ED15/ED16 and ED16/ED17. For each boundary, a quasibinomial model containing developmental phase and stimulation condition was fitted, and relative model fit was evaluated using residual deviance. The ED14/ED15 boundary yielded the lowest residual deviance among the five candidate two-phase models and was therefore selected as the exploratory two-phase representation (Supplementary Table S07). This model, comprising an early phase (ED12–ED14) and late phase (ED15–ED17), was compared with the additive model containing all six embryonic-day groups using an analysis-of-deviance F-test to assess the loss of fit associated with reducing age to two phases (Supplementary Table S08). A Phase × Stimulation interaction was additionally examined to determine whether the estimated phase difference varied according to stimulation condition (Supplementary Tables S09, S12). Because the phase boundary was selected from the observed data, this analysis was considered exploratory, and associated p-values were interpreted descriptively.

## Supplementary Information

Supplementary Information 1.

Supplementary Information 2.

Supplementary Information 3.

## Data Availability

All data analysed during this study are included as a raw data in the supplementary material.
