# Supplementary material for: Hypothalamic c-Fos expression changes during late chicken embryonic development and increases following experimental stimulation
Source: Sci Rep. 2026 Sep 5;16:27817. doi: 10.1038/s41598-026-65626-x (PMC13546284; doi:10.1038/s41598-026-65626-x)
Supplement: Supplementary file 1 [file 41598_2026_65626_MOESM2_ESM.docx]

| ***Table S01: Type II ANOVA – Additive Model (Agegroup + Stimulus)*** | | | |
| --- | --- | --- | --- |
| **Term** | **Df** | **F** | **p** |
| Agegroup | 5 | 13,06 | <0.001 |
| Stimulus | 1 | 123,41 | <0.001 |
| Residuals | 65 |  |  |

**Supplementary Table S01: Type II ANOVA – Additive Model (Agegroup + Stimulus)**

Results of a Type II ANOVA F-test applied to the additive quasibinomial GLM, including main effects of Agegroup and Stimulus.

| ***Table S02:*** ***Estimated Marginal Means – Full Age Group Model (Agegroup + Stimulus)*** | | | | | |
| --- | --- | --- | --- | --- | --- |
| **Agegroup** | **Stimulus** | **prob** | **SE** | **lower.CL** | **upper.CL** |
| ED12 | ctrl | 0,0001 | 0,0001 | 0 | 0,0004 |
| ED13 | ctrl | 0,0001 | 0,0001 | 0 | 0,0004 |
| ED14 | ctrl | 0,0002 | 0,0001 | 0,0001 | 0,0006 |
| ED15 | ctrl | 0,0007 | 0,0003 | 0,0003 | 0,0017 |
| ED16 | ctrl | 0,0005 | 0,0002 | 0,0002 | 0,0013 |
| ED17 | ctrl | 0,0009 | 0,0004 | 0,0004 | 0,0022 |
| ED12 | stim | 0,0018 | 0,0011 | 0,0006 | 0,0059 |
| ED13 | stim | 0,0024 | 0,0011 | 0,0009 | 0,006 |
| ED14 | stim | 0,0051 | 0,0014 | 0,0029 | 0,0089 |
| ED15 | stim | 0,0149 | 0,003 | 0,01 | 0,0222 |
| ED16 | stim | 0,0108 | 0,0026 | 0,0067 | 0,0175 |
| ED17 | stim | 0,0197 | 0,0026 | 0,0151 | 0,0256 |

**Supplementary Table S02: Estimated Marginal Means – Full Age Group Model (Agegroup + Stimulus)**

Estimated marginal means (EMMs) derived from the full six-stage additive quasibinomial GLM for each combination of Agegroup and Stimulus. prob: EMM on the response scale (predicted proportion of c-Fos-positive cells); lower.CL and upper.CL: 95% confidence interval bounds. Source data for Fig. 2 and Fig. S3. CL, Confidence Level.

| ***Table S03: Type II ANOVA – Interaction Model (Agegroup × Stimulus)*** | | | |
| --- | --- | --- | --- |
| **Term** | **Df** | **F** | **p** |
| Agegroup | 5 | 12,595 | <0.001 |
| Stimulus | 1 | 119,046 | <0.001 |
| Agegroup:Stimulus | 5 | 0,146 | 0.981 |
| Residuals | 60 |  |  |

**Supplementary Table S03: Type II ANOVA – Interaction Model (Agegroup × Stimulus)**

Results of a Type II ANOVA F-test applied to the interaction quasibinomial GLM, including main effects of Agegroup and Stimulus and their interaction term (Agegroup × Stimulus).

| ***Table S04 – Model Comparison – Interaction vs. Additive*** | | | | | |
| --- | --- | --- | --- | --- | --- |
| **Model** | **Resid_Df** | **Resid_Dev** | **Df** | **F** | **p** |
| Agegroup + Stimulus (additiv) | 65 | 865,7 |  |  |  |
| Agegroup * Stimulus (with interaction) | 60 | 854,3 | 5 | 0,1459 | 0.981 |

**Supplementary Table S04: Model Comparison – Interaction vs. Additive**

F-test-based comparison of the additive and interaction quasibinomial GLMs, assessing whether inclusion of the Agegroup × Stimulus interaction term significantly improves model fit.

| ***Table S05 – Pairwise Tukey-adjusted Contrasts (All Age Groups)*** | | | | | | |
| --- | --- | --- | --- | --- | --- | --- |
| **contrast** | **odds.ratio** | **SE** | **df** | **null** | **t.ratio** | **p.value** |
| ED12 / ED13 | 0,762 | 0,574 | 65 | 1 | -0,361 | 0.999 |
| ED12 / ED14 | 0,355 | 0,233 | 65 | 1 | -1,58 | 0.614 |
| ED12 / ED15 | 0,12 | 0,075 | 65 | 1 | -3,378 | 0.015 |
| ED12 / ED16 | 0,166 | 0,107 | 65 | 1 | -2,796 | 0.071 |
| ED12 / ED17 | 0,091 | 0,055 | 65 | 1 | -3,944 | 0.003 |
| ED13 / ED14 | 0,466 | 0,251 | 65 | 1 | -1,417 | 0.717 |
| ED13 / ED15 | 0,158 | 0,08 | 65 | 1 | -3,661 | 0.006 |
| ED13 / ED16 | 0,218 | 0,114 | 65 | 1 | -2,915 | 0.053 |
| ED13 / ED17 | 0,119 | 0,057 | 65 | 1 | -4,424 | 0.001 |
| ED14 / ED15 | 0,339 | 0,116 | 65 | 1 | -3,155 | 0.028 |
| ED14 / ED16 | 0,468 | 0,173 | 65 | 1 | -2,055 | 0.324 |
| ED14 / ED17 | 0,255 | 0,079 | 65 | 1 | -4,426 | 0.001 |
| ED15 / ED16 | 1,383 | 0,436 | 65 | 1 | 1,027 | 0.907 |
| ED15 / ED17 | 0,754 | 0,182 | 65 | 1 | -1,166 | 0.851 |
| ED16 / ED17 | 0,546 | 0,151 | 65 | 1 | -2,184 | 0.259 |

**Supplementary Table S05: Pairwise Tukey-adjusted Contrasts (All Age Groups)**

All 15 pairwise contrasts between embryonic age groups (ED12–ED17), derived from the additive quasibinomial GLM and marginalised over stimulus condition. Contrasts are expressed as odds ratios on the log-odds scale. P-values are Tukey-adjusted for multiple comparisons. Results form the basis for the compact letter display in Supplementary Table S05.

| ***Table S06: Estimated Marginal Means and Compact Letter Display (Age Groups)*** | | | | |
| --- | --- | --- | --- | --- |
| **Agegroup** | **prob** | **lower.CL** | **upper.CL** | **letter** |
| ED12 | 0 | 0 | 0,001 | a |
| ED13 | 0,001 | 0 | 0,001 | a |
| ED14 | 0,001 | 0,001 | 0,002 | a |
| ED15 | 0,003 | 0,002 | 0,006 | b |
| ED16 | 0,002 | 0,001 | 0,004 | ab |
| ED17 | 0,004 | 0,003 | 0,007 | b |

**Supplementary Table S06: *Estimated Marginal Means and Compact Letter Display (Age Groups)***

Estimated marginal means (EMMs) derived from the additive quasibinomial GLM, marginalised over stimulus condition, for each embryonic age group (ED12–ED17). prob: EMM on the response scale (predicted proportion of c-Fos-positive cells); lower.CL and upper.CL: 95% confidence interval bounds. Compact letter display (CLD) summarises pairwise comparisons from Supplementary Table S05; age groups sharing a letter do not differ significantly. Source data for Fig. 3. CL, Confidence Level.

| ***Table S07: Residual Deviance by Phase Model Cut-point*** | |
| --- | --- |
| **Cutoff** | **Deviance** |
| ED12/ED13 | 1598,3 |
| ED13/ED14 | 1260,2 |
| ED14/ED15 | 1008,5 |
| ED15/ED16 | 1370,4 |
| ED16/ED17 | 1354,1 |

**Supplementary Table S07: Residual Deviance by Phase Model Cut-point**

Residual deviance of the quasibinomial GLM fitted separately for each tested binary phase cut-point, used to identify the optimal developmental phase boundary.

| ***Table S08: Model Comparison – Phase Model vs. Full Age Group Model*** | | | | | |
| --- | --- | --- | --- | --- | --- |
| **Model** | **Resid_Df** | **Resid_Dev** | **Df** | **F** | **p** |
| Phase (ED14/ED15) + stimulus | 69 | 1008,5 |  |  |  |
| Agegroup (6 levels) + stimulus | 65 | 865,7 | 4 | 2,356 | 0.063 |

**Supplementary Table S08: Model Comparison – Phase Model vs. Full Age Group Model**

F-test-based comparison of the two-phase model (Phase + Stimulus) and the full six-stage additive model (Agegroup + Stimulus, see Supplementary Table S01), assessing the adequacy of the phase-based grouping.

| ***Table S09: Type II ANOVA – Phase Model (Phase* × *Stimulus)*** | | | |
| --- | --- | --- | --- |
| **Term** | **Df** | **F** | **p** |
| Phase | 1 | 43,51 | <0.001 |
| Stimulus | 1 | 100,44 | <0.001 |
| Phase:Stimulus | 1 | 0,04 | 0.847 |
| Residuals | 68 |  |  |

**Supplementary Table S09: Type II ANOVA – Phase Model (Phase × Stimulus)**

Results of a Type II ANOVA F-test applied to the two-phase quasibinomial GLM, including main effects of Phase and Stimulus and their interaction term (Phase × Stimulus).

| ***Table S10: Raw Image Analysis Data*** | | | | | |
| --- | --- | --- | --- | --- | --- |
| **Sample** | **Agegroup** | **Stimulus** | **cFos** | **Histone_H3** | **cFos_rel_pct** |
| 20240108-3C | ED12 | stim | 2 | 4035 | 0,049566 |
| 20240205-15A1 | ED12 | stim | 22 | 6095 | 0,360952 |
| 20240205-16A2 | ED12 | stim | 11 | 4137 | 0,265893 |
| 20240205-17A2 | ED12 | stim | 7 | 4581 | 0,152805 |
| 20240205-18A2 | ED12 | stim | 0 | 4021 | 0 |
| 20240115-1B | ED12 | ctrl | 1 | 3366 | 0,029709 |
| 20240115-2C | ED12 | ctrl | 0 | 3737 | 0 |
| 20240115-3C | ED12 | ctrl | 0 | 5678 | 0 |
| 20240115-4C | ED12 | ctrl | 0 | 4918 | 0 |
| 20240109-1C | ED13 | stim | 0 | 4223 | 0 |
| 20240109-2C | ED13 | stim | 0 | 4435 | 0 |
| 20240109-4D | ED13 | stim | 0 | 3468 | 0 |
| 20240206-14A2 | ED13 | stim | 17 | 4254 | 0,399624 |
| 20240206-15A2 | ED13 | stim | 20 | 4411 | 0,453412 |
| 20240206-16A2 | ED13 | stim | 17 | 4206 | 0,404184 |
| 20240206-17A2 | ED13 | stim | 10 | 4087 | 0,244678 |
| 20240116-1C | ED13 | ctrl | 3 | 3371 | 0,088994 |
| 20240116-2C | ED13 | ctrl | 0 | 4822 | 0 |
| 20240116-3A | ED13 | ctrl | 0 | 4111 | 0 |
| 20240116-4C | ED13 | ctrl | 4 | 3836 | 0,104275 |
| 20240110-1A | ED14 | stim | 12 | 4617 | 0,259909 |
| 20240110-2A | ED14 | stim | 13 | 4969 | 0,261622 |
| 20240110-3A | ED14 | stim | 114 | 4954 | 2,301171 |
| 20240110-4A | ED14 | stim | 7 | 5427 | 0,128985 |
| 20240221-13A | ED14 | stim | 31 | 4464 | 0,694444 |
| 20240221-1A | ED14 | stim | 7 | 4176 | 0,167625 |
| 20240221-2A2 | ED14 | stim | 4 | 4340 | 0,092166 |
| 20240221-4A1 | ED14 | stim | 3 | 3831 | 0,078309 |
| 20231030-1A2 | ED14 | ctrl | 0 | 7383 | 0 |
| 20231030-2A2 | ED14 | ctrl | 0 | 6269 | 0 |
| 20231030-3A3 | ED14 | ctrl | 4 | 6760 | 0,059172 |
| 20240117-1D | ED14 | ctrl | 0 | 5151 | 0 |
| 20240117-2D | ED14 | ctrl | 0 | 4043 | 0 |
| 20240117-3C | ED14 | ctrl | 2 | 3871 | 0,051666 |
| 20240117-4D | ED14 | ctrl | 0 | 6444 | 0 |
| 20231107-2C | ED15 | stim | 143 | 6739 | 2,121977 |
| 20240111-1C | ED15 | stim | 39 | 4454 | 0,875617 |
| 20240111-2C2 | ED15 | stim | 105 | 5094 | 2,061249 |
| 20240221-14A1 | ED15 | stim | 41 | 4280 | 0,957944 |
| 20240221-15A2 | ED15 | stim | 23 | 3167 | 0,726239 |
| 20231031-1C3 | ED15 | ctrl | 2 | 6774 | 0,029525 |
| 20231031-2D | ED15 | ctrl | 0 | 6435 | 0 |
| 20231031-4D | ED15 | ctrl | 0 | 6149 | 0 |
| 20240118-1C | ED15 | ctrl | 1 | 4041 | 0,024746 |
| 20240118-2A | ED15 | ctrl | 11 | 4793 | 0,229501 |
| 20240118-4A | ED15 | ctrl | 7 | 4273 | 0,163819 |
| 20240118-3A | ED15 | ctrl | 7 | 3385 | 0,206795 |
| 20231108-1D2 | ED16 | stim | 30 | 3743 | 0,801496 |
| 20231108-2B | ED16 | stim | 80 | 6959 | 1,14959 |
| 20240321-5B1 | ED16 | stim | 11 | 2205 | 0,498866 |
| 20240321-7B1 | ED16 | stim | 9 | 1997 | 0,450676 |
| 20240325-2B1 | ED16 | stim | 89 | 4607 | 1,931843 |
| 20240325-4C | ED16 | stim | 26 | 3012 | 0,863214 |
| 20231024-3D | ED16 | ctrl | 3 | 5914 | 0,050727 |
| 20231024-4B | ED16 | ctrl | 7 | 6441 | 0,108679 |
| 20231024-5B | ED16 | ctrl | 2 | 7533 | 0,02655 |
| 20240321-3A3 | ED16 | ctrl | 1 | 4339 | 0,023047 |
| 20240321-8B2 | ED16 | ctrl | 1 | 5624 | 0,017781 |
| 20231109-1E1 | ED17 | stim | 179 | 7316 | 2,446692 |
| 20231109-2E | ED17 | stim | 102 | 6084 | 1,676529 |
| 20240219-10B1 | ED17 | stim | 57 | 4910 | 1,160896 |
| 20240219-11A1 | ED17 | stim | 35 | 6351 | 0,551094 |
| 20240219-14A1 | ED17 | stim | 229 | 5259 | 4,35444 |
| 20240219-4A1 | ED17 | stim | 40 | 3716 | 1,076426 |
| 20240326-6A1 | ED17 | stim | 48 | 3721 | 1,289976 |
| 20240326-8A1 | ED17 | stim | 148 | 5137 | 2,881059 |
| 20231025-1E | ED17 | ctrl | 2 | 4985 | 0,04012 |
| 20231025-3E | ED17 | ctrl | 11 | 6899 | 0,159443 |
| 20240219-5A2 | ED17 | ctrl | 1 | 4467 | 0,022386 |
| 20240219-6A1 | ED17 | ctrl | 4 | 5370 | 0,074488 |
| 20240219-8A2 | ED17 | ctrl | 3 | 5634 | 0,053248 |
| 20231025-2E | ED17 | ctrl | 8 | 6400 | 0,125 |

**Supplementary Table S10: Raw Image Analysis Data**

Individual-level output of the FIJI macro pipeline. Sample: unique sample identifier. Agegroup: embryonic day (ED12–ED17). Stimulus: stimulation condition ("stim" = experimental stimulation; "ctrl" = control). cFos: number of detected c-Fos-positive cells. Histone_H3: number of detected Histone H3-positive cells. cFos_rel_pct: proportion of c-Fos-positive cells relative to Histone H3-positive cells (%). This table constitutes the source data for all statistical analyses. cFos_rel_pct values correspond to the individual data points shown in Fig. S3.

| ***Table S11: Model Coefficients – Additive Model (Agegroup + Stimulus)*** | | | | |
| --- | --- | --- | --- | --- |
| **Term** | **Estimate** | **SE** | **t** | **p** |
| (Intercept) | -9,3739 | 0,7264 | -12,904 | <0.001 |
| AgegroupED13 | 0,2716 | 0,7527 | 0,361 | 0.719 |
| AgegroupED14 | 1,0361 | 0,6558 | 1,58 | 0.119 |
| AgegroupED15 | 2,1189 | 0,6273 | 3,378 | 0.001 |
| AgegroupED16 | 1,7948 | 0,6418 | 2,796 | 0.007 |
| AgegroupED17 | 2,4008 | 0,6087 | 3,944 | <0.001 |
| Stimulusstim | 3,0638 | 0,4335 | 7,068 | <0.001 |

**Supplementary Table S11: Model Coefficients: Additive Model (Agegroup + Stimulus)**

Raw model coefficients of the additive quasibinomial GLM on the log-odds scale, including standard errors, t-values, and p-values for each predictor level.

***Table S12: Estimated Marginal Means***

| **Phase** | **prob** | **SE** | **lower.CL** | **upper.CL** |
| --- | --- | --- | --- | --- |
| early | 0,0007 | 0,0002 | 0,0004 | 0,0014 |
| late | 0,0035 | 0,0009 | 0,0021 | 0,0057 |

**Supplementary Table S12: *Estimated Marginal Means – Phase Model (Phase + Stimulus)***

Estimated marginal means (EMMs) derived from the two-phase quasibinomial GLM for each combination of Phase and Stimulus. prob: EMM on the response scale (predicted proportion of c-Fos-positive cells); lower.CL and upper.CL: 95% confidence interval bounds. CL, Confidence Level. Source data for Fig. 4.

| ***Table S13: Software and Package Versions*** | |
| --- | --- |
| **Package** | **Version** |
| (Fiji Is Just) ImageJ | 2.16.0 |
| Java | 1.8.0_322 [64-Bit] |
| R | 4.5.2 |
| emmeans | 2.0.3 |
| multcompView | 0.1.11 |
| multcomp | 1.4.30 |
| car | 3.1.5 |
| ggsignif | 0.6.4 |
| tidyverse | 2.0.0 |
| scales | 1.4.0 |
| writexl | 1.5.4 |

**Supplementary Table S13: Software and Package Versions**

Overview of Software and R Packages used for image analysis and statistical modelling, including version numbers.
