# Supplementary material for: Hypothalamic c-Fos expression changes during late chicken embryonic development and increases following experimental stimulation
Source: Sci Rep. 2026 Sep 5;16:27817. doi: 10.1038/s41598-026-65626-x (PMC13546284; doi:10.1038/s41598-026-65626-x)
Supplement: Supplementary file 2 [file 41598_2026_65626_MOESM3_ESM.docx]

**Fig. S1: Representative immunofluorescence images of the paraventricular nucleus (PVN) in control embryos across embryonic days ED14–ED17.** "Merged (raw)" shows the unprocessed fluorescence images, whereas binary images of c-Fos (green) and Histone H3 (red) were generated by FIJI during image post-processing. Histone H3 staining was consistent across groups, as in Fig. 1. No c-Fos-positive cells were detected in the representative sections shown, consistent with the low baseline levels of c-Fos expression observed in control embryos. Section size: 464.98 × 464.98 µm; scale bar: 200 µm.

**Fig. S2: Representative immunofluorescence images of the paraventricular nucleus (PVN) in stimulated and control embryos at early developmental stages (ED12–ED13).** "Merged (raw)" shows the unprocessed fluorescence images, whereas binary images of c-Fos (green) and Histone H3 (red) were generated by FIJI during image post-processing. Histone H3 staining was consistent across groups, as in Fig. 1. No c-Fos-positive cells were detected in the representative sections shown, consistent with the low baseline levels of c-Fos expression observed in younger embryos. Section size: 464.98 × 464.98 µm; scale bar: 200 µm.

**Fig. S3: c-Fos expression varies across embryonic development and with experimental stimulation condition: raw data overlay.** Individual data points are shown alongside estimated marginal means. Individual data points represent the relative proportion of c-Fos-positive cells per animal (%). Estimated marginal means and 95% CIs are derived from the quasibinomial GLM and are shown on the same scale. Source data are provided as a Source Data file
